# The Effect of Biopolymer Chitosan on the Rheology and Stability of Na-Bentonite Drilling Mud

**DOI:** 10.3390/polym13193361

**Published:** 2021-09-30

**Authors:** Basim Abu-Jdayil, Mamdouh Ghannam, Karam Alsayyed Ahmed, Mohamed Djama

**Affiliations:** Chemical & Petroleum Engineering Department, U.A.E. University, Al-Ain P.O. Box 15551, United Arab Emirates; mamdouh.ghannam@uaeu.ac.ae (M.G.); 201270114@uaeu.ac.ae (K.A.A.); 200935432@uaeu.ac.ae (M.D.)

**Keywords:** bentonite, chitosan, biopolymer, drilling mud, rheology, yield stress, thixotropy

## Abstract

The utilization of greens resources is a grand challenge for this century. A lot of efforts are paid to substitute toxic ingredients of the conventional drilling mud system with nontoxic natural materials. In this paper, the effect of the natural polymer chitosan on the rheology and stability of sodium-bentonite drilling mud was investigated in the polymer concentration range of 0.1–3.0 wt.%. Both the shear and time dependent rheological properties of pure chitosan, pure bentonite and bentonite–chitosan dispersions were studied. Moreover, zeta potential measurements were used to evaluate the stability of bentonite-chitosan suspension. Adding chitosan improved the natural properties of drilling mud, namely: yield stress, shear thinning, and thixotropy. The viscosity of bentonite suspension increased significantly upon the addition of chitosan in the concentration range of 0.5 to 3.0 wt.% forming network structure, which can be attributed to the interactions of hydrogen bonding between -OH clusters on the bentonite surface with the NH group in the chitosan structure. On the other hand, dispersed chitosan–bentonite suspension was observed at low chitosan concentration (less than 0.5 wt.%). Increasing both bentonite and chitosan concentrations led to the flocculation of the bentonite suspension, forming a continuous gel structure that was characterized by noteworthy yield stress. The desired drilling mud rheological behavior can be obtained with less bentonite by adding chitosan polymer and the undesirable effects of high solid clay concentration can be avoided.

## 1. Introduction

The use of natural minerals in different industrial applications has recently received special attention because of their ease of availability, abundance in nature, environmentally-friendly properties and low cost [1]. Bentonite is one of the valuable natural minerals, which contains mainly of montmorillonite and varying quantities of other minerals like quartz and feldspar. Although there are many bentonite types, the most important one is the sodium bentonite, which has a characteristic high swelling capacity [2].

With unique rheological behavior, bentonites are used in many branches of industry, including drilling fluids, paper, cement, dyes, pharmaceuticals, nanocomposites, polymer composites, and ceramics (e.g., [3,4,5,6,7,8,9]). Because bentonite has a porous structure with a high capability of adsorption, it was used extensively in the removal of different pollutants such as heavy metals and organic pollutants (e.g., [10,11,12]).

One of the important applications of Na-bentonite suspensions is their use as drilling fluids in the oil and gas industry [13,14,15]. Carrying the rock cuttings to the surface is one of the functions of drilling muds, which needs a minimum yield stress. In addition, drilling muds are used to maintain a sufficient pressure against the rock formation, which can be accomplished by the high viscosity of the mud. Moreover, drilling muds are applied to lubricate and cool the bit, where enough fluidity that comes from the mud shear thinning behavior is needed [16].

Sodium bentonite is used extensively in drilling operations as its dispersions from colloidal material with exaptational rheological properties. Small amounts of Na–bentonite (2.0–9.0 wt.%) in water can form a viscous, shear thinning fluid with remarkable yield stress [17,18,19]. High-viscosity behavior accompanied with yield stress at low shear rates is denoting solids carrying capacity away from the drill bit, and shear thinning behavior (low viscosity at high shear rate) in the neighborhood of the drill bit is needed to minimize torque requirements. Moreover, dispersions of sodium bentonite showed a thixotropic behavior at high enough solid concentration [17,20,21,22,23]. 

Many challenges face the drilling engineers such as developing a drilling mud that yields good well stability with minimum fluid loss. Therefore, different additives, mainly polymers and surfactants, are utilized with bentonite suspensions to modify the rheological behavior of the suspensions to meet the specification of the desired application, such as poly vinyl pyrolidone [24], poly vinyl alcohol [25,26], xanthan [27], polyanionic cellulose [28], polyethyleneimine [29], poly (ethylene glycol) [30], polypropylene glycol [31] and carboxymethyl cellulose sodium salt [32,33], polyacrylamide [34], polyethyleneimine [35], cetyltrimethyl ammonium bromide (CTAB) and sodium dodecyl sulphate (SDS) [36,37]. In addition, the reduction of bentonite concentration in the drilling mud is another objective of the drilling engineers to avoid the undesirable effects of high solid concentrations that lead to a reduction in the rate at which a wellbore can be drilled to a given depth. Two other major concerns on the high solid content of drilling muds are the high cost of transportation and storage, which can be significant for drilling sites located in distant and hostile environments [38].

Two grand challenges for this century are renewable energy and the utilization of green resources. Nowadays, the preparation of drilling fluids and their additives is a difficult task, considering both the technical and environmental factors [39]. Recently, much attention has been paid to develop low cost, sustainable, environmentally friendly, and high-performance water-based drilling muds. There are a lot of efforts in replacing toxic ingredients from conventional drilling fluid system by a truly nontoxic natural substitute(e.g., [39,40,41]). 

Chitin is a natural polysaccharide, which is the second most abundant natural polymer in the world. Chitosan is a derivative of chitin that can be prepared by deacetylation of chitin in an alkaline medium [42]. Both chitin and chitosan are renewable, biodegradable, environmentally friendly, and bio functional materials that come from natural resources and waiting for a market [42]. However, the poor solubility of chitin is the major limiting factor in its utilization and the investigation of its properties and structure [43]. Important properties of chitosan include solubility in various media, high viscosity, polyelectrolyte behavior, polyoxysalt formation, the ability to form films, metal chelations, optical, and structural characteristics [44] and applications are including but not limited to cosmetics, water engineering, textile industry, food processing, agriculture, chromatographic separations, solid state batteries, biomedical applications such as tissue engineering, and even photography [45,46].

Currently, many adsorption methods using chitosan composites are being developed to adsorb dyes and other pollutants as a substitute for conventional waste-water treatment methods [12,47,48]. In the open literature, the modification of the bentonite dispersions with chitosan has received little attention. In this study, the effect of chitosan biopolymer on the rheological properties of Na–bentonite suspensions was investigated in the polymer concentration range of 0.1–3.0 wt.%. Both the shear and time dependent rheological properties of bentonite–chitosan dispersions were studied. In addition, the stability of bentonite suspensions was evaluated by measuring the zeta potential.

## 2. Materials and Methods

### 2.1. Materials

Bentonite used in this work was provided by Sigma-Aldrich CHEMIE GmbH, Germany. The composition of bentonite sample is shown in Table 1. The bentonite used is classified as Na–bentonite with ${\mathrm{Na}}^{+}{/\mathrm{Ca}}^{+2}$ ratio of 3.73. The average particle size of bentonite was 5.34 µm.

The chitosan (molecular weight 8000–12,000) used in this work was provided by the Jordanian Pharmaceutical Manufacturing Co. (Naor, Jordan) and was obtained from Qingdao Rich Ocean Industrial Co., Ltd., Shandong, China.

### 2.2. Suspension Preparation

In this study, two solid concentrations, 4.0 and 8.0 wt.%, of bentonite dispersions were prepared. The 4.0 wt.% concentration is within the range of bentonite concentrations that are often used in the formulation of drilling mud and, the second, 8.0 wt.%, was chosen as the more general for other applications of bentonite dispersions. These two solid concentrations were selected to represent different rheological behaviors of bentonite dispersions [33]. The 4.0 and 8.0 wt.% bentonite dispersions were labelled in this work by B4 and B8, respectively. In order to avoid the formation of aggregates and ensure a homogeneous system, dispersions were prepared by adding bentonite slowly to deionized water under continuous magnetic stirring conditions. 

An appropriate amount of chitosan was added to deionized water containing 1.0 vol.% acetic acid under continuous stirring conditions to prepare the chitosan solution. The stirring was stopped when the solute was completely dissolved. The solutions of desired 0.1, 0.2, 0.5, 1.0, 2.0, and 3.0 wt.% were prepared.

Bentonite–chitosan dispersions were prepared by taking the bentonite suspension and the chitosan solution prepared separately and then adding the chitosan solution slowly to the bentonite under stirring conditions. Then, the prepared dispersions were poured into a covered container and kept at room temperature for specific time.

### 2.3. Rheological Measurements

Rheolab QC viscometer from Anton Paar, Germany, was used to measure the rheological properties of prepared suspensions. The measuring system coaxial cylinder was used in the study according to ISO 3219 and DIN 53019. The radii of the measuring cup and were 14.460 mm and 13.329 mm, respectively. While the cone angle was 120°. This geometry allows a gap width of 1.132 mm. The flow curves of the prepared dispersion were determined by measuring the shear stress (τ) of the samples as a function of shear rate ($\dot{\gamma }$) at a constant temperature. The flow curves were determined in the shear rate range of 2.0 to 1500 $s^{-1}$ This range of shear rate was selected to cover wide range of industrial applications. Among those is the drilling fluid which is usually characterized at shear rate of 511 and 1022 $s^{-1}$ [49]. All rheological tests were performed at 25 °C ± 0.1. The rheological measurements were performed by increasing (forward measurements) and decreasing (backward measurements) shear rates. The area confined between the upward flow curve and downward flow curve was calculated as a measure for mud thixotropy using data analysis option of RHEOPLUS/32 V3.31 software. The rheological test was performed at 24 h after sample preparation and prior to measurement, the sample was stirred in the viscometer for 1 min at a shear rate of 5 $s^{-1}$, followed by a rest time for 2 min.

## 3. Results and Discussion

### 3.1. Rheology of Pure Chitosan

**Figure 1:**
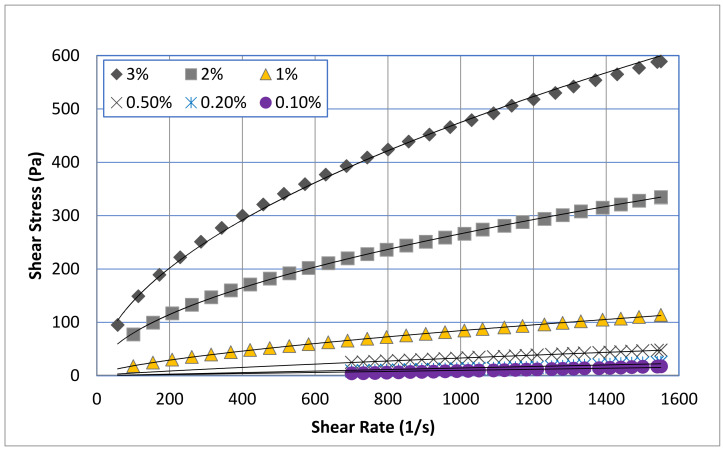
Flow curves of pure chitosan solution at different concentrations fitted with power-law model.

The effect of concentration on the flow curves of pure chitosan solutions is presented in Figure 1. This work covers a wide range of chitosan concentrations of 0.10 to 3.0 wt.%. It is clear that the shear stress values have clear dependence on chitosan concentration and shear rate. Shear stress gradually increases by increasing the chitosan concentration and shear rate. The effect of chitosan concentration is more remarkable at high shear rate. Modelling analysis according to the Herschel–Bulkely model (Equation (1)) was accomplished for all chitosan solutions:

(1) $$\tau =\tau_{0}+m{\dot{\gamma }}^{n}$$

where τ is the shear stress, $\tau_{0}$ is the yield stress, $\dot{\gamma }$ is the shear rate, *n* is the flow behavior index and *m* is the consistency coefficient. However, it was found that the power law model (Equation (2)), which is a special case of Herschel–Bulkely model with $\tau_{0}$ = 0, fits very well all the tested solutions of chitosan. The solid lines on Figure 1 represent the plot of the power-law model, and the regressed parameters (*m* and *n*) are reported in Table 2.

(2) $$\tau =m{\dot{\gamma }}^{n}$$

**Figure 2:**
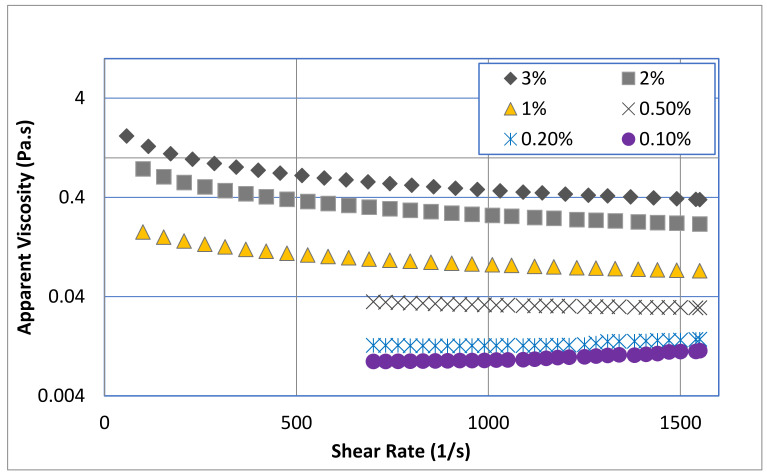
Effect of shear rate on the apparent viscosity of pure chitosan solution at different concentrations.

As can be seen from Figure 2, low concentrations of chitosan solutions up to 0.2 wt.% showed Newtonian behavior (*n* =1), with constant apparent viscosity with shear rate. Increasing the chitosan concentration beyond 0.5 wt.% shifted the rheological behavior to non-Newtonian, as the apparent viscosity of chitosan solution decreases with shear rate. The analysis of the flow curves revealed that the chitosan at high concentrations exhibited a shear thinning behavior without yield stress. Table 2 shows a clear decrease in the flow behavior index (*n*) from unity (i.e., Newtonian behavior) at 0.2 wt.% chitosan concentration to 0.53 (i.e., shear thinning behavior) at 3.0 wt.% concentration. On the other hand, increasing the chitosan concentration resulted in a significant increase in the consistency coefficient (*m*), which is a measure of the material viscosity, from 0.01 Pa·s^n^ at 0.1 wt.% chitosan concentration to 12.04 Pa·s^n^ at 3.0 wt.% chitosan solution. The same rheological trend was observed by other researchers [50,51,52,53].

The apparent viscosity increased notably with an increase in chitosan concentration (Figure 2), implying the formation of a transient network within the experimental concentration range. From the data reported in Table 2, the relationship between the consistency coefficient (*m*) of chitosan and the solution concentration can be illustrated by a power law formula:

(3) $$m=0.947conc.{\left(wt.\%\right)}^{2.27}$$

where conc is the chitosan concentration in wt.%. The interpretation of this relationship that at very dilute chitosan solutions, the polymer chains are widely separated without remarkable overlaps. At a critical concentration demonstrating the transition from the very dilute to the dilute concentration region, the hydrodynamic volumes of individual polymer chains begin to touch. As the solution concentration is increased further, the chains begin to overlap, finally resulting in the entanglements formation that notably increase viscosity. The exponent of the *m* and concentration relationship was approximately equal to two, indicating consistency coefficient (viscosity) was caused by “two-body” collisions. Wang et al. [53] attributed this to the increased connecting points of chitosan aggregates at a high concentration, corresponding to the high apparent viscosity.

Many researchers (e.g., [52,54,55,56,57,58]) reported stronger shear thinning behavior of chitosan solution at high concentrations. The shear thinning behavior was attributed to the amine groups of chitosan protonate in acidic solution which limits the hydrophobic interaction and hydrogen bonding between the polymeric chains retains chitosan in solution form, resulting in typical polymeric solution behavior (i.e., shear thinning behavior). Applied shear exposes the charged groups and due to the electrostatic and steric repulsions viscosity decreases continuously with shear (Figure 2) [57]. The increase of the non-Newtonian behavior of chitosan solutions with concentration was attributed to the chain-expanding structure and consequent increase in entanglement. A recent investigation by Taherian et al. [59] reported that the shear thinning behavior of chitosan depends on chitosan molecular weight and solution concentration. They found that any increase in concentration of chitosan causes a decrease in flow behavior index (*n*) and an increase in consistency coefficient, *m*, owing to the lower hydrodynamic interactions in dilute solution and the close contact of chains which constrains the flow of concentrated solutions. 

**Figure 3:**
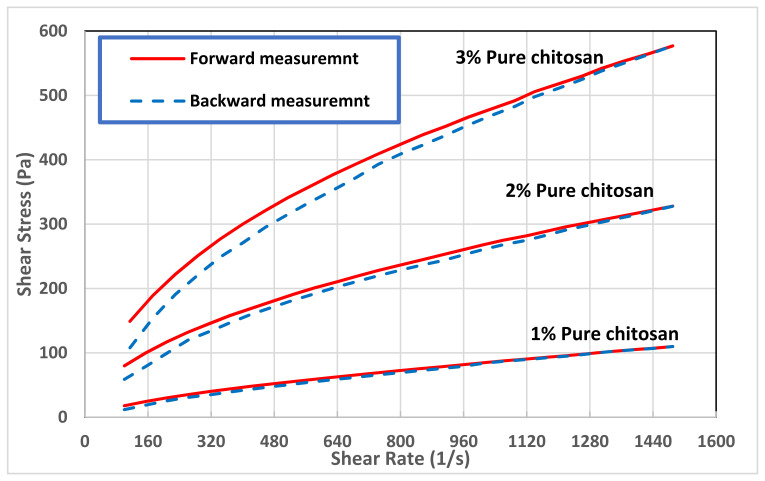
Hysteresis loops of pure chitosan solution at different concentrations.

On the other hand, the time-dependent behavior of chitosan solutions was investigated by performing gradual increase in the shear rate (forward measurement), while reporting the values of shear stress, followed by a gradual decrease in the shear rate (backward measurement). The presence of hysteresis loops, the difference between the forward and backward measurements, reveals that the suspension showed a thixotropic behavior. The time dependent behavior was detected in chitosan solutions with concentrations higher than 1.0 wt.%, see Figure 3. Obviously, the Newtonian materials such as the 0.1 and 0.2 wt.% chitosan solutions do not exhibit time-dependent behavior. In addition, the 0.5 wt.% chitosan solution showed negligible hysteresis loop, which was within the error range of the shear stress values. The area of the hysteresis loop reported in Table 2 as well as shown in Figure 3 reveal that the thixotropic behavior of chitosan solution increased significantly with chitosan concentration. This behavior may be explained by the alignment of the polymeric chains induced by the flow. At higher concentrations, the effect of this alignment becomes more evident, intensely changing the shear stress required for a given deformation [50]. It should be highlighted here that if the viscosity falls and immediately returns after it has stopped shearing, the material is not thixotropic material but just shear thinning, see for example the behavior of high concentration solutions illustrated in Figure 2.

### 3.2. Rheology of Pure Bentonite

**Figure 4:**
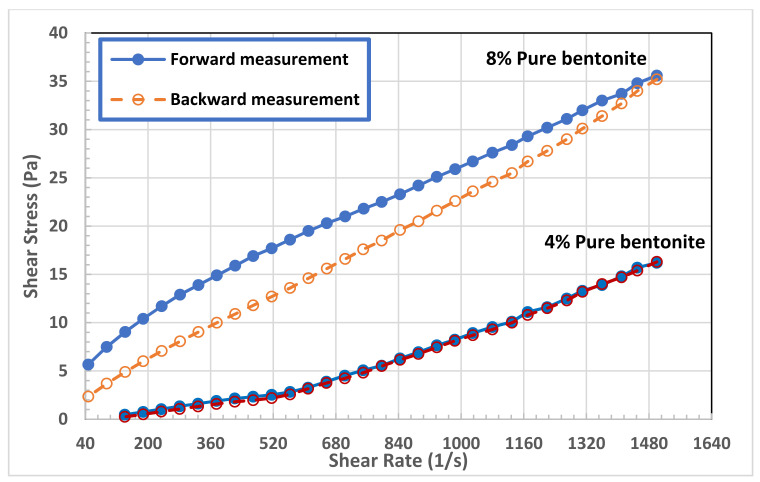
Flow curves of bentonite dispersions at 4.0 and 8.0 wt.%.

The rheological behavior of pure sodium bentonite was reported in detail for solid concentrations of 0.5–10.0 wt.% in the work of Abu-Jdayil [17]. The rheological behaviors of the pure bentonite with 4.0 and 8.0 wt.% solid content are shown in Figure 4. Both bentonite dispersions showed non-Newtonian behavior at most of the tested range of shear rate. Modelling analysis of the samples’ flow curves predicted that the Herschel–Bulkley model (Equation (1)) fits well with all the measured data of the bentonite suspensions. The regressed parameters of the Herschel–Bulkley model that are reported in Table 3, show that the 4.0 wt.% bentonite dispersion (B4) behaved like a Bingham fluid in shear rate range of 47–560 $s^{-1}$, which switched into shear thickening material (n>1.0) in the shear rate range of 560–1500 $s^{-1}$ without remarkable yield stress. On the other hand, the 8.0 wt.% bentonite suspension (B8) exhibited shear thinning behavior (n<1.0) with a noticable yield stress. It is projected that the solid concentration will bring about an increase in all rheological properties such as the yield stress $\tau_{0}\;$ and the consistency coefficient (*m*). At a high bentonite concentration (like 8.0 wt.%), it is expected that flocculation will produce a continuous networked structure instead of individual flocs that generate in lower concentration solution. In such kind of dispersions, the network structure builds up slowly with time, as the particles orient themselves towards positions of minimum free energy under the influence of Brownian motion [13].

The time-dependent behavior of bentonite suspensions is also illustrated in Figure 4. The presence of hysteresis loops in flow curves indicates that the bentonite dispersion exhibited a time-dependent behavior. Figure 4 shows that the backward shear stress values are lower than that of the forward measurement for the B8 bentonite dispersion, which means that this sample exhibited a clear thixotropic behavior, where the viscosity decreased with shearing time. On the other hand, the thixotropic behavior of B4 dispersion was not clear. In addition, the area of hysteresis loops reported in Table 3 for both bentonite concentrations shows that the degree of thixotropy of B8 samples is orders of magnitude greater than that of B4 sample. The thixotropic behavior is a typical rheological behavior of many clay dispersions, which is concentration dependent. The degree of thixotropy of sodium bentonite increased considerably with solid concentration [17]. The fragments of the network structure which are broken under shear, need time to be restructured again to a three-dimensional network [13]. The network structure is broken by high shear stress and interparticle bonds tend to re-form themselves with time. The occurrence of thixotropy in a material means that the rate of bond breakdown dominates the rate of rebuilding process.

### 3.3. Rheology of Bentonite-Chitosan Dispersions

**Figure 5:**
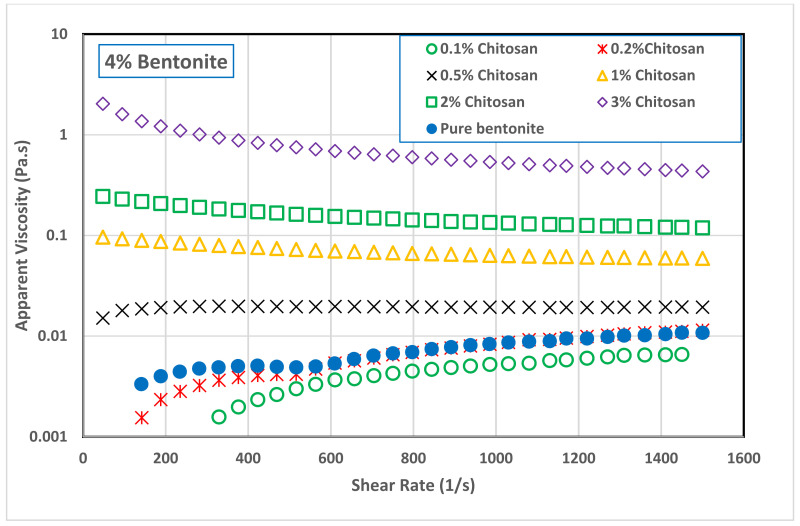
Effect of chitosan concentration on the viscosity of 4.0 wt.% bentonite dispersion.

**Figure 6:**
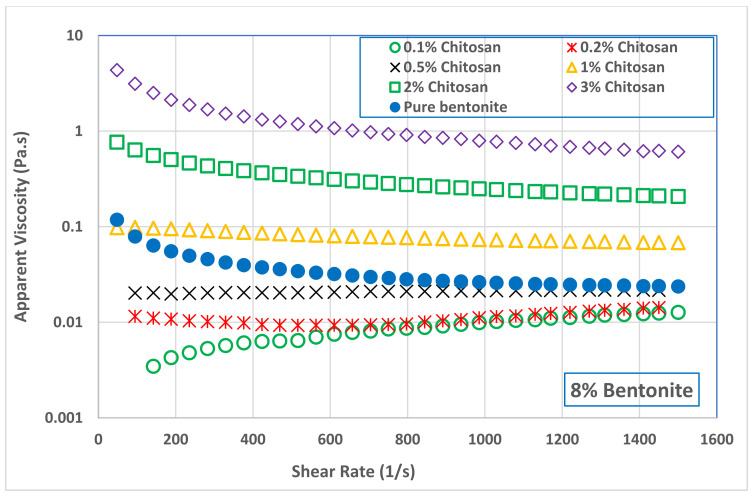
Effect of chitosan concentration on the viscosity of 8.0 wt.% bentonite dispersion (B8).

Addition of low concentration of chitosan (0.1 and 0.2 wt.%) to B4 decreased slightly the suspension apparent viscosity comparing with pure bentonite suspension. Figure 5 shows that increasing the chitosan concertation above 0.5 wt.% increased significantly the viscosity of B4 suspension. A similar behavior was also observed with B8 suspension, as seen in Figure 6. Addition of small amounts of chitosan to B8 suspension led to a slight reduction in its viscosity. However, this reduction was followed by a significant increase in B8 viscosity as the chitosan concentration increased in the range of 0.5 to 3.0 wt.%. At the shear rate of 511 s^−1^ (where the drilling mud is usually tested), adding 3.0 wt.% of chitosan to bentonite suspensions increased the viscosity of B4 sample from 4.98 to 720 mPa s while increased the viscosity of B8 samples from 34 to 1188 mPa s. In general, it can be concluded that the network structure of bentonite–chitosan suspension builds up slowly with chitosan concentration to form a shear thinning behavior. In addition, noticeable yield stress at high bentonite and chitosan concentration was observed. This network structure is because of the interaction of hydrogen bonding between –OH clusters on the surface of bentonite with an –NH–group in the chitosan structure.

**Figure 7:**
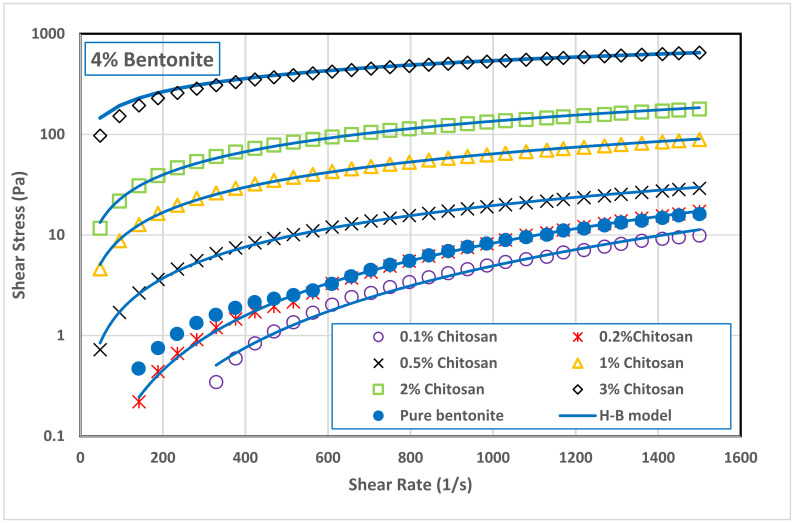
Effect of chitosan concentration on the flow curves of 4.0 wt.% bentonite dispersion.

**Figure 8:**
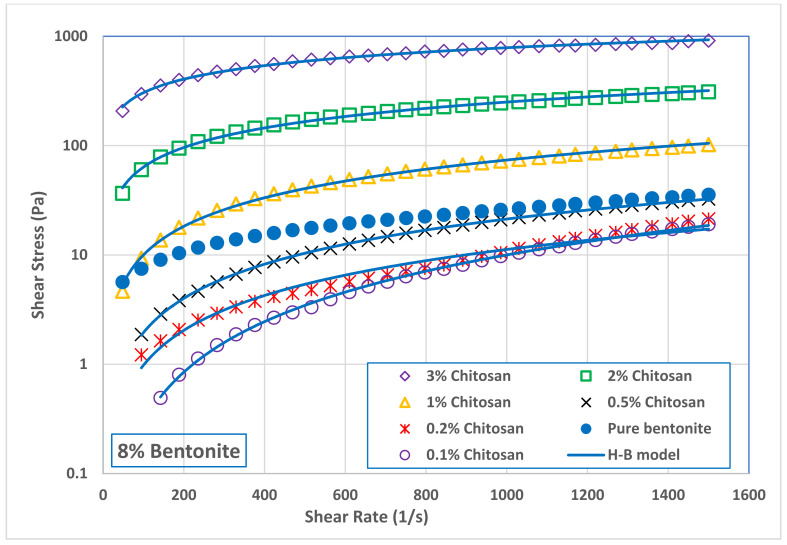
Effect of chitosan concentration on the flow curves of 8.0 wt.% bentonite dispersion.

The initial reduction in bentonite viscosity upon the addition of a low quantity of chitosan means that the biopolymer chains made some kind of disturbance in the network structure of bentonite dispersion. In other words, it can be a result of the collapse of chitosan molecules together with flocculated bentonite particles. This network disturbance was reflected on the flow curves of both bentonite samples, B4 and B8, illustrated in Figure 7 and Figure 8, and on their rheological parameters reported in Table 3. At low chitosan concentration (0.1 and 0.2 wt.%,) the rheological behavior of B4 suspension was shear thickening (n> 1.0). This behavior is turned into Newtonian behavior (n~1.0) at 0.5 wt.% chitosan concentration, followed by shear thinning behavior (n< 1.0) at a chitosan concentration of 1.0 wt.% and above. On the other hand, the B8 suspension exhibited a shear thickening behavior at 0.1 wt.% chitosan concentration, which is then inverted into Newtonian behavior at 0.2 and 0.5 wt.% chitosan, and to shear thinning behavior at chitosan concentrations beyond 1.0 wt.%. Table 3 shows that increasing both the bentonite and chitosan concentration significantly increased the consistency coefficient, m (which is a reflection of viscosity value), and decreased the flow behavior index, *n* (i.e., increasing the shear thinning behavior). On the other hand, adding chitosan biopolymer to B8 suspension initially removed its yield stress, which appeared again at high chitosan concentration, namely, at 2.0 and 3.0 wt.%. The main advantage of chitosan addition is that the desired suspension rheological behavior can be obtained with less solid content by adding chitosan polymer and the undesirable effects of high solid bentonite concentrations can be avoided. The apparent viscosity of 8.0 wt.% pure bentonite dispersions was reached by adding less than 1.0 wt.% chitosan to 4.0 wt.% bentonite dispersions, compare Figure 5 and Figure 6. In addition, the combined flow behaviors of Bingham and shear thickening observed for the B4 pure bentonite dispersion was shifted toward the shear thinning behavior in the presence of the chitosan.

The significant increase in shear stress and viscosity of the bentonite dispersion upon the addition of chitosan in the concentration range of 0.5–3.0 wt.% can be attributed to the adhering of chitosan molecules on the surface of clay particles and swelling behavior in slurry. The increase of the dispersion viscosity is an indication of forming a network structure within the dispersion. The charged nature of chitosan (positive due to nitrogen bonding) is noted to cause the viscosity to increase, which was observed in other systems like kaolin [60] and rice flour formulation [46], where chitosan addition considerably increased the viscosity of both materials. At the conditions used in this study, it is expected that the chitosan is adsorbed on bentonite particles via hydrogen bonding of carbonyl and/or hydroxyl groups from chitosan macromolecules to hydroxyl groups present on the surface of bentonite, as well as via electrostatic interactions between $NH_{3}^{+}$ groups from chitosan and negative sites present on the surface of bentonite. Azzam et al. [61] reported that chitosan intercalates with bentonite silicate layer by $NH_{2}$ groups.

On the other hand, Table 3 shows that adding chitosan to B4 and B8 eliminated their yield stress, which appeared later in suspensions at high concentrations of chitosan (2.0 and 3.0 wt.%). The disappearance of the yield stress from most of the B4-chitosan suspensions indicates that the bentonites particles are weekly dispersed in the chitosan solution. Increasing both the bentonite and chitosan concentrations shifted the suspension from weekly dispersed to flocculated suspension which demonstrated a significant value of yield stress; 16.5 Pa and 25.5 Pa for B4 and B8 in 3.0 wt.% chitosan solution, respectively. The repulsion between negatively charged bentonite particles is highest in pure water (because the diffuse double layers of the particles interfere), resulting in a dispersed suspension. If a polymer like chitosan is added, the diffuse double layer compresses and particles can approach each other. In general, different modes of particle association may occur. This particle association, and particularly the edge-to-face (EF) and edge-to-edge (EE) associations, lead to flocculate the bentonite suspension, forming a continuous gel structure that is characterized by a yield stress.

In the drilling practice, high values of yield stress for a drilling mud are an indication of a good cuts carrying capacity. On the other hand, the higher the yield stress, the higher the pressure required to restart circulation after a certain period of stagnation during the drilling operation. Therefore, a very high value for the yield point is not desirable. The yield stress values exhibited by bentonite suspensions in 2.0 and 3.0 wt.% chitosan solution (15.2–25.5 Pa) are comparable with the reported values of oilfield drilling fluid [23] and within the specifications of the American Petroleum Institute for drilling fluids [62].

**Figure 9:**
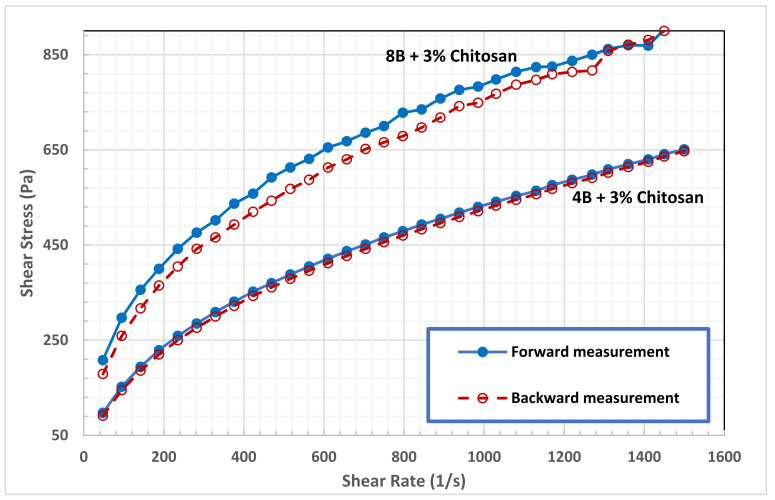
Effect of 3.0 wt.% of chitosan on Hysteresis loops B4 and B8 suspensions.

**Figure 10:**
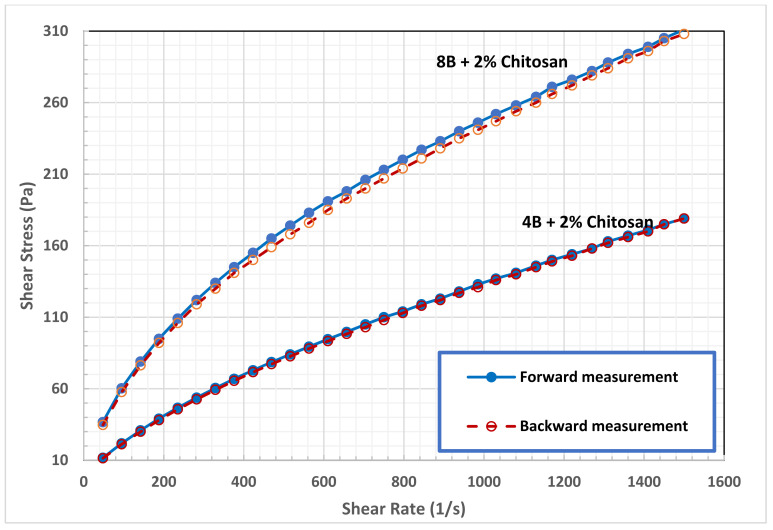
Effect of 2.0 wt.% of chitosan on hysteresis loops B4 and B8 suspensions.

The thixotropic behavior of bentonite suspension, which is a favorable property for drilling fluid [20], was enhanced substantially at a high concentration of chitosan, as seen in Figure 9 and Figure 10. Thixotropic behavior occurs because the fragments of the network which are broken under shear, need time to be linked again in a three-dimensional network. The presence of thixotropy means that the rate of bond breaking is greater than that required for the rebuilding process [17]. As reported in Table 3, the hysteresis loops area of B4 and B8 in 3.0 wt.% chitosan solution demonstrating the strong thixotropic nature of the drilling fluid. Comparing to pure suspension, the hysteresis area of B4 increased by 177% and 1995% in 2.0 and 3.0 wt.% chitosan solutions, respectively. While the hysteresis area of B8 increased by 95% and 1285%, in the same chitosan solution. The results of this work can support the hypothesis that although thixotropy and yield stress are separated phenomena, they show a tendency toward appearing in the same fluid. Table 3 shows that the same bentonite–chitosan suspensions had clear yield stress, and exhibited clear thixotropic behavior. Indeed, it is believed that the yield stress and thixotropy is caused by the same fundamental physics [20]. The same microstructure present in a fluid that resists large rearrangements (which is responsible for the yield stress), when broken by flow, is believed to be the origin of thixotropy. However, the structure developed at high bentonite and chitosan concentrations is readily broken under shear. This structure may be classified as a fragile gel; one that forms a weak associative network that breaks easily under flow.

### 3.4. Stability of Bentonite-Chitosan Dispersions

**Figure 11:**
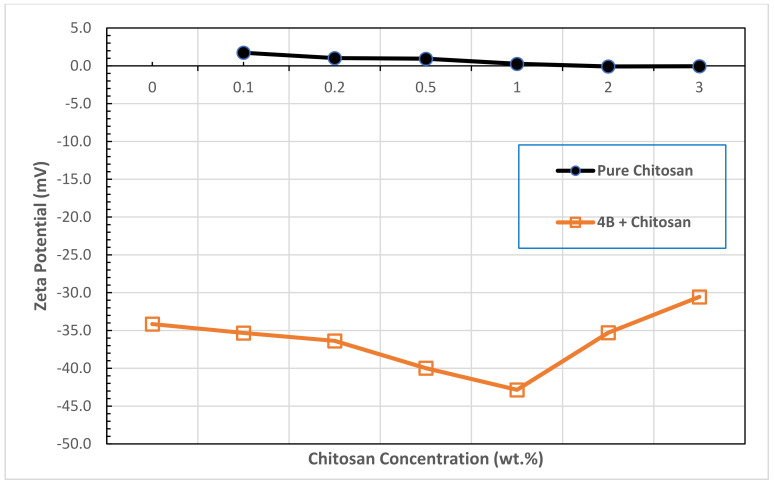
Zeta potential of pure chitosan and B4-chitosan suspension.

Finally, the stability of bentonite–chitosan suspension was evaluated by measuring the zeta potential. The magnitude of the zeta potential indicates the degree of electrostatic repulsion between adjacent, similarly charged particles in dispersion. Zeta potential values for pure chitosan solution and for bentonite–chitosan suspensions are plotted in Figure 11. It is clear that the zeta potential for chitosan solution slightly decreases with increasing the chitosan concentration to almost zero mV. Similar results were also reported in the literature for chitosan at around pH 6.0 [63] indicating the loss of protonated amino groups. As can be seen in Figure 11, zeta potential values of bentonite–chitosan suspension are negative and in the range of −30.46 mV to −42.85 mV, indicating the formation of stable suspension. It was indicted that clay particles having the zeta potential values higher than +30 mV or lower than −30 mV, are forming as stable suspensions [64]. However, adding the chitosan solution in the concentration range of 0.1 to 1.0 wt.%, to the bentonite dispersions led to increase the absolute value of the zeta potential, suggesting that the clay particles became more dispersed, this behavior was accompanied with the loss of the yield stress as found in the previous sections. The further increase of chitosan concentration slightly decreased the zeta potential indicating that the dispersion became more flocculated. This result correlates with the trend of viscosity increase and the appearance of the yield stress at high chitosan concentration.

## 4. Conclusions

In this work, the rheology and stability of bentonite dispersions modified by different concentrations of biopolymer chitosan were investigated. From the experimental work and analytical modeling performed in this study, we arrived at the following conclusions.

The apparent viscosity of pure chitosan increased notably with an increase in chitosan concentration, implying the formation of a transient network within the experimental concentration range.

Both detected shear thinning, and thixotropic behaviors of pure chitosan increased with concentration. The shear thinning behavior was attributed to the amine groups of chitosan protonate in acidic solution which limits the hydrophobic interaction and hydrogen bonding between the polymeric chains retains chitosan in solution form, resulting in typical polymeric solution behavior.

Adding chitosan in the concentration range of 0.5 to 3.0 wt.% to bentonite suspensions significantly increased its viscosity. It can be concluded that the network structure of bentonite–chitosan suspension builds up slowly with chitosan concentration to form a shear thinning behavior with noticeable yield stress at high bentonite and chitosan concentration. This network structure is because of the interaction of hydrogen bonding between –OH clusters on the surface of bentonite with an –NH group in the chitosan structure.

The yield stress values exhibited by bentonite suspensions in 2.0 and 3.0 wt.% chitosan solution (15.2–25.5 Pa) are comparable with the reported values of oilfield drilling fluid and within the specifications of the American Petroleum Institute for drilling fluids.

The hysteresis loops area of bentonite–chitosan suspensions demonstrated the strong thixotropic nature of the drilling fluid. In addition, the results of this work can support the hypothesis that although thixotropy and yield stress are separated phenomena, they show a tendency toward appearing in the same fluid.

Zeta potential values of bentonite–chitosan suspensions were negative and in the range of −30.46 mV to −42.85 mV, indicating the formation of stable suspension. Increasing both the bentonite and chitosan concentrations shifted the suspension from weekly dispersed to flocculated suspension which demonstrated a significant value of yield stress.

One of the main advantages of chitosan addition is that the desired suspension rheological properties can be obtained with less bentonite by adding chitosan polymer and the undesirable effects of high solid bentonite concentrations can be avoided. The viscosity of 8.0 wt.% pure bentonite dispersions can be achieved by adding less than 1.0 wt.% chitosan to 4.0 wt.% bentonite dispersions.

## Figures and Tables

**Table 1:** Chemical composition of bentonite sample in wt%.

Composition	${\mathrm{Na}}_{2}O$	$K_{2}O$	${\mathrm{Fe}}_{2}O_{3}$	MgO	${\mathrm{Al}}_{2}O_{3}$	${\mathrm{SiO}}_{2}$	CaO	FeO	H_2_O	Trace Elements
wt%	2.425	0.245	3.250	2.670	21.080	63.020	0.650	0.350	5.64	0.720

**Table 2:** Power-law regressed parameters for chitosan solutions.

Chitosan Conc. (wt.%)	$\dot{\gamma }$ Range (s^−1^)	*m* (Pa·s^n^)	*n*	A (Hysteresis Loop Area) (Pa/s)
0.10	700–1550	0.0104	1.0	-
0.20	700–1550	0.0143	1.0	-
0.50	700–1550	0.1075	0.83	-
1.00	100–1550	0.8792	66	3202
2.00	100–1550	7.1727	0.52	11,330
3.00	57.2–1550	12.0380	0.53	24,240

**Table 3:** Herschel–Bulkley parameters and area of hysteresis loops of bentonite dispersions at different chitosan concentrations.

Solid Conc. (wt.%)	Chitosan Conc. (wt.%)	$\dot{\gamma }$ Range (s^−1^)	$\tau_{0}$ (Pa)	*m* (Pa·s^n^)	*n*	A (Hysteresis Loop Area) (Pa/s)
4.0	0.0	47.8–560	1.60	0.0033	1.0	577
	0.0	560–1500	0.0	0.0001	1.7	
	0.1	329–1500	0.0	$4\times 10^{-6}$	2.0446	165
	0.2	142–1500	0.0	$3\times 10^{-5}$	1.8085	179
	0.5	47.8–1500	0.0	0.0154	1.0348	252
	1.0	47.8–1500	0.0	0.2011	0.8344	912
	2.0	47.8–1500	0.0	0.6979	0.7824	1600
	3.0	47.8–1500	16.50	21.7080	0.4608	12.090
8.0	0.0	47.8–1500	8.65	0.0652	0.82	3189
	0.1	142–1500	0.0	0.0003	1.533	3005
	0.2	94.7–1500	0.0	0.0075	1.058	1255
	0.5	94.7–1500	0.0	0.0164	1.038	1178
	1.0	47.8–1500	0.0	0.1842	0.8676	4853
	2.0	47.8–1500	15.2	5.1782	0.5552	6223
	3.0	47.8–1500	25.50	64.772	0.3583	44,140

## Data Availability

Not applicable.
